# Health Information Exchange: Contributions from 2024

**DOI:** 10.1055/s-0046-1816623

**Published:** 2026-11-03

**Authors:** Sue S. Feldman, Meryl Bloomrosen

**Affiliations:** 1 Graduate Programs in Health Informatics, Department of Health Services Administration, University of Alabama at Birmingham, Birmingham, ALUSA; 2 Department of Biomedical Informatics and Data Science, Heersink School of Medicine, University of Alabama at Birmingham, Birmingham, ALUSA; 3 American Medical Association, Chicago, ILUSA

**Keywords:** International Medical Informatics Association Yearbook, Health Information Exchange, Health Information, Interoperability, Data Sharing, Health Information Technology, Electronic Health Records, Heath Data Governance, Health Data Standards

## Abstract

**Objectives** : To summarize the recent literature and research and present a selection of the best papers published online and in print in 2024 related to Health Information Exchange (HIE) and Health Information Systems (HIS).

**Method** : Using Covidence as a screening and analysis tool, a systematic review of the literature was independently conducted by the two HIE section editors. Ten studies emerged as suitable for final IMIA Yearbook consideration.

**Results** : Among the papers reviewed, there were far fewer papers about technology and infrastructure, and more papers on the use of HIE/HIS by clinicians and health professionals, and discussions about barriers and facilitators related to HIE/HIS use. Of the three papers selected across unique countries (Japan, US, and Nepal), there were eight cross-cutting themes.

**Conclusions** : Review of the literature revealed more international studies this year, suggesting perhaps a maturing, but not yet fully mature, of the HIE/HIS infrastructure and governance in other countries and/or increased access to systems and data for research. In terms of study locations, only half of the final 10 papers were US based. Considering the larger corpus of literature reviewed and the changing landscape of electronic health record systems in the US (i.e., especially consolidation in the acute care hospital marketplace with a few dominant vendors), section editors have discussed the potential for expanded and/or more robust HIE and the broader HIS landscape, including vendor-specific HIEs/HIS. Globally, there is increased consideration of open data standards, citizen's rights and controls over one's data, and concerns about data provenance and data governance. Additionally, health data exchange and sharing, privacy and security, governance, and public policy create a persistent tension between what is possible and what is achieved regarding domestic, international, and intercontinental health data exchange. As AI finds more uses within health data, increased governance may be warranted to decrease friction between privacy and security vs. data sharing.

## 1. Introduction

Review of the 2024 literature notes that health information exchange (HIE) internationally, is viewed across the broader landscape of health information “systems” rather than “exchanges”, suggesting management of healthcare beyond electronic health records (EHRs) and beyond clinical data [ 1 ]. In fact, the World Health Organization (WHO) suggests that the health information systems (HIS), which include HIEs, cover all levels from community/facility to national with feedback from each domain to contribute to the strengthening of health service performance [ 2 ]. Interestingly, WHO has not bridged this level internationally, but rather describes this framework as siloed within a country. This represents a larger area of HIE, or HIS, expansion over the next decade, especially as countries adopt EHR systems that are more than clinical data repositories and contain their own internal method of exchanging data. It is important to note that even though EHR adoption from the same vendors across the globe may occur, data harmonization may still persist between these EHR systems or within systems from which data are consumed into the EHR, such as those used in laboratory systems (i.e., Logical Observation Identifiers Names and Codes - LOINC) and medication libraries (i.e., RxNorm) [ 3 ].

Electronic exchange of medical information across healthcare settings – historically known as HIE – enables the rapid, secure movement of patient data between disparate providers and institutions [ 4 5 ]. These systems are typically supported by centralized or collaborative entities that oversee the governance and implementation of the exchange infrastructure and transactions. The core aim has historically been to support informed clinical decisions by improving timely access to patient data, thereby fostering care that is safer, more effective, and more equitable [ 1 , 5 6 7 ]. However, HIEs are not simply contained within a healthcare setting, but rather have found their utility across care environments and locations, including at the community level [ 8 ]. Furthermore, the Organization for Economic Cooperation and Development (OECD) has called for digital transformation of healthcare systems in OECD countries to support quality healthcare and reduce barriers to care [ 9 ]. Given the global implementations of diverse approaches to health data collection and potential convergence of terminology such as HIE and HIS terminology, we use both terms together in this discussion, as HIE/HIS, and do not distinguish between HIE as in exchange of medical information across healthcare settings and HIS as in a larger and more mature ecosystem of medical information, health-related, as opposed to healthcare settings, and individual systems.

## 2. About the Paper Selection

In January 2025, with the assistance of a medical librarian, the co-editors conducted a PubMed and Embase search using MeSH headings, keywords, and synonyms in titles and abstracts with a focus on HIE/HIS. The search was country agnostic, but the publication must have been written in English. The publication year included first online and print publications between January 1, 2024 and December 31, 2024, inclusive. The search strategy is shown in Table 1 .

**Table 1.** Search strategy in PubMed and Embase.

Cross-cutting Theme	Best Papers		
	Japan [ 19 ] (Suzumoto et al.)	US [ 18 ] (Snyder et al.)	Nepal [ 13 ] (Bhatt et al.)
Access not equal to Effective use	X	X	X
Usability and Technical Barriers	X	X	X
Variation in Usage Patterns	X	X	X
Training and Capacity Gaps	X	X	X
Data Quality and Trust Issues	X	X	X
Policy and Infrastructure Gaps	X	X	X
Local Context and Governance		X	X
Missed Opportunities	X	X	X

## 3. Outlook

The ten candidate best papers for 2024 reflected an increase in attention toward actual connections or what happens as a result of the connection, instead of the ability to connect, and suggest that HIE/HIS is at a critical juncture with a focus toward clinical users, their perspectives, and the uses of HIE/HIS in the clinical space. Across the ten papers, five themes emerged: data quality and completeness, interoperability and fragmentation, human factors and usability, governance and policy, and health equity. While technical capabilities have advanced significantly (i.e., the ability to connect), the human, organizational, economic, and regulatory issues may present challenges with outcomes (i.e., what happens after implementation).

The tension between the promise of interoperability and the reality of fragmented, burdensome systems persists. Success appears to require not only technical solutions, but fundamental changes to workflows, incentive structures, governance, user training, and regulatory frameworks. Additionally, the role of AI in reducing this tension cannot be underscored enough. For example, the HIE section survey paper by Vest *et al.* highlights the potential for AI to offer promising methodologies across HIE/HIS to facilitate and accelerate insights related to some of healthcare's most vexing issues, such as utilization of services and emergency department overcrowding, and also to expand models to impact chronic care delivery [ 10 ]. That being said, the documentation burden revealed by Holmgren *et al.* serves as an important reminder that adding more technical capabilities without addressing underlying workflow issues may actually worsen, rather than improve, the very issues trying to be solved [ 11 ].

### 3.1 Data Quality and Completeness

High-quality, complete data are foundational to the effective functioning of HIS. Across the reviewed literature, data quality, defined as the accuracy, timeliness, consistency, and completeness of health records [ 12 ], emerges as both a technical and governance challenge. Several studies reveal concerns around data completeness and accuracy [ 13 14 15 ]. Bhatt *et al.* discuss that adequate training, data elements at the point of collection, and power and internet were often barriers to data completeness [ 13 ].

By contrast, and more of a US perspective, Ha *et al.* noted that data completeness was contingent on integration across providers, legal clarity on access, and data format standardization [ 16 ]. Improvements in data completeness not only improve system reliability, but also uncover hidden disparities. For example, the paper by Coelho *et al.* found that the proportion of missing race/color data declined from 34.7% to 21.2% over the study period, resulting in a correlation between data completeness and visibility of racial disparities in hospitalizations [ 14 ]. It is important to recognize that technical completeness (e.g., presence of data fields) does not guarantee functional completeness (data used for care) and that underutilization, fragmentation, and poor design often result in perceived incompleteness, even when data exist.

### 3.2 Interoperability and Fragmentation

Interoperability, the capacity of disparate HIS to exchange, interpret, and use data, is a foundational principle in achieving seamless, patient-centered care. Fragmentation, its counterpoint, reflects disconnected information flows, duplicated effort, and clinical inefficiencies. Dixon *et al.* used C-CDA documents from two major HIE/HIS sources (regional and vendor-based), to suggest that there were discrepancies in available patient data, low concordance between data sources, and fragmented data domains (e.g., labs present in one source, medications in another) [ 15 ]. These findings underscore that interoperability between HIEs/HIS is limited and that structural fragmentation exists even within technologically advanced systems.

The study by Matthews *et al.* explored a critical interoperability gap: the limited data exchange between behavioral health and general medical providers [ 17 ]. This study suggests that legal and organizational silos persist, with only 33% of providers in advanced delivery models (e.g., patient-centered medical homes, accountable care organizations), engaging in behavioral health HIE/HIS, limiting interprofessional data integration. The study by Sasie *et al.* reminds us that public health must remain part of the discussion around HIE/HIS [ 21 ]. Even when stakeholders recognized the need for and relevance of data exchange for public health emergencies, fragmentation can undermine coordinated response efforts. As described by others, fragmentation can be brought about by poor integration among stakeholder institutions [ 15 18 ], incompatible data element formats [ 15 17 ], infrastructure gaps [ 17 ], and financial and cultural barriers to data sharing [ 17 19 ].

While great strides have been made toward interoperability and decreased fragmentation, technical heterogeneity across platforms [ 15 19 ], fragmented governance and policy environments [ 16 , 20 ], siloed data ecosystems, especially in behavioral and emergency care [ 16 20 ], and poor user integration and workflow alignment [ 11 18 ] are still reported in the literature as key barriers.

### 3.3 Human factors and usability

Across the studies, six usability barriers were identified: inadequate training [ 13 21 ], poor interface design [ 18 19 ], trust [ 15 18 ], organizational alignment [ 20 ], legal and ethical concerns [ 16 ], and crisis-related stress, and as in public health emergencies, in which usability failures may be amplified during a crisis [ 21 ].

Relative to training, the studies suggest that system usability is undermined by inadequate user training, lack of onsite coaching, and limited availability of learning materials and that success depends not just on system deployment, but on equipping users with the skills and motivation to engage effectively [ 13 21 ]. Additionally, two papers expand poor interface design as a general barrier to include more specific details such as visual clutter and complex navigation that discourage system use—even when data are available, as contributors to usability [ 18 19 ].

Usability is not just about function, but about how intuitive and user-friendly systems are in real-world settings. As such, success depends not just on system deployment but on equipping users with the skills and motivation to engage effectively. From a broader perspective, systems must be embedded into daily tasks, not layered on top. Doing so, may balance compliance and usability to avoid cognitive overload.

The literature is replete with studies on documentation burden. While many studies tend to link documentation burden with quality of clinician life, the study by Holmgren *et al.* provides evidence that each additional hour of documentation reduced HIE/HIS use by 7.1% [ 11 ].

While the studies for 2024 focused on usability from a clinical or high technical literacy level perspective, the study by Ha *et al.* sets forth new usability challenges as platforms and uses shift toward patient-focused use [ 16 ]. These are considerations for developers to be aware of and to ensure that human factors and user centered design principles are adhered to. Usability is a prerequisite for system success and investments in infrastructure must be aligned with investments in training, interface design, and workflows.

### 3.4 Governance and Policy

Governance and policy are the scaffolding that enables or inhibit the implementation, integration, and impact of HIS. Strong governance ensures data quality, stakeholder coordination, accountability, equity, and sustainability. Across the reviewed studies, governance structures and policy frameworks emerge as central determinants of both system adoption and long-term viability.

More specifically, across the ten studies, five governance barriers were identified: voluntary participation [ 15 19 20 ], regularity overload [ 11 ], lack of local capacity [ 13 21 ], legal ambiguity [ 16 18 ], and fragmented coordination [ 17 21 ]. Moreover, policy implications were identified relative to the governance barriers. For example, from an international perspective, strengthening governance sponsorship with targeted funding and mandate-based accountability [ 13 19 ], strengthening regulatory compliance and data use for equity monitoring [ 14 ], and establishing unified legal and policy frameworks that allow governance space for innovation [ 16 ] and emergency response [ 21 ] were all recognized as contributing to governance structure and policy framework needs.

While the policy implications across the US studies may suggest a bit more HIE/HIS maturity, they were not too dissimilar for those in the international literature… just more mature. For example, Dixon *et al.* suggested that national HIE/HIS standards need better enforcement [ 15 ], more specifically, incentives for participation in TEFCA (Trusted Exchange Framework and Common Agreement) as a mechanism to close existing standard gaps [ 20 ] and provide for breaking down organizational silos [ 18 ]. Much of the HIE/HIS governance in the US is out of office of the Assistant Secretary for Technology Policy and Office of the National Coordinator for Health Information Technology (ASTP/ONC) (formally ONC), and, in fact, Holmgren *et al.* suggest that federal rule-making can go a long way toward streamlining documentation requirements such that current regulatory overload undermines system usability and function [ 11 ]. While there has been some relaxation of 43 Code of Federal Regulations part 2 in the behavioral health space, this was investigated specifically by Matthews *et al.* with the suggestion that delivery reforms lack explicit enforcement for behavioral health HIE/HIS, possibly due to the lack of expectations for behavioral health exchange [ 17 ].

Regardless of the maturity level, aligning policies across the local, national, and transnational spectrum, especially in federated or cross-border systems, could provide an environment that facilitates and fosters broader exchange of health information, resulting in better healthcare and better health outcomes, truly agnostic to geography.

### 3.5 Health Equity

Health equity in the context of HIE/HIS refers to the fair and just access to health data, digital tools, and the benefits they confer, regardless of social, geographic, economic, or demographic factors. Across the ten studies, equity emerges as a latent, but increasingly critical, dimension, especially where data incompleteness, underutilization, and structural disparities converge. For example, at a very fundamental and foundational level is the issue of access, with suggestions that rural and behavioral health domains need targeted support and investments to facilitate access and use [ 11 13 17 19 20 21 ]. As such, it might be helpful to understand HIS through a health equity lens in terms of various inequities (geographic [ 13 19 21 ], organizational [ 11 15 20 ], etc.) and domain usage (rural [ 13 19 21 ], behavioral [ 17 ], emergencies [ 21 ]) to lower barriers to data use and exchange. Rarely is health equity investigated relative to HIS or presented with any level of specificity; these papers present an opportunity to do so.

### 3.6 Papers for Inclusion

With increased specificity as a focus, we took a closer look at the resulting three papers selected for being best papers for 2024 Yearbook HIE section ( Table 2 ). Across the papers, there were eight cross-cutting themes: access, usability, usage patterns, training and capacity, data quality and trust, policy and infrastructure gaps, local context and governance, and missed opportunities ( Table 3 . Cross-cutting themes in selected papers). These papers are summarized in the Appendix section.

**Table 2.** Best papers selected for the 2025 IMIA Yearbook of Medical Informatics for the section Health Information Exchange. The articles are listed in alphabetical order of the first author's surname.

Section Health Information Exchange
• Bhatt PR, Bhandari R, Adhikari S, Gahatraj NR. Health professionals' experience on district health information system (DHIS2) and its utilization at local levels in Gandaki province, Nepal: a qualitative study. PLOS Global Public Health. 2024 Mar 27;4(3):e0002890. • Snyder ME, Nguyen KA, Patel H, Sanchez SL, Traylor M, Robinson MJ, Damush TM, Taber P, Mixon AS, Fan VS, Savoy A. Clinicians' use of Health Information Exchange technologies for medication reconciliation in the US Department of Veterans Affairs: a qualitative analysis. BMC Health Services Research. 2024 Oct 8;24(1):1194. • Suzumoto J, Mori Y, Kuroda T. Health Care Worker Usage of Large-Scale Health Information Exchanges in Japan: User-Level Audit Log Analysis Study. JMIR Medical Informatics. 2024 Oct 9;12(1):e56263.

**Table 3.** Cross-cutting themes in selected papers

PubMed
(“Health Information Exchange”[Majr] OR Health-Information-Exchange*[Title/Abstract] OR Medical-Information-Exchange*[Title/Abstract] OR Clinical-information-system* OR clinical-pharmacy-information-system*[Title/Abstract] OR health-information-network*[Title/Abstract] OR health-information-system*[Title/Abstract] OR medical-information-service*[Title/Abstract] OR is-h-med[Title/Abstract]) AND ("2024/01/01"[Date - Publication] : "2024/12/31"[Date - Publication]) AND (("Artificial Intelligence"[Majr] OR "Computer Security"[Majr] OR "Medical Informatics"[Majr] OR "Natural Language Processing"[Majr] OR AI[tiab] OR artificial-intelligence[tiab] OR clinical-informatics[tiab] OR computational-intelligence[tiab] OR computer-reasoning[tiab] OR computer-security[tiab] OR computer-vision-system*[tiab] OR cyber-security[tiab] OR cybersecurity[tiab] OR health-informatics[tiab] OR Health-Information-Technolog*[tiab] OR HIT[tiab] OR machine-intelligence[tiab] OR machine-learning[tiab] OR medical-computer-science*[tiab] OR medical-informatics[tiab] OR medical-information-science*[tiab] OR natural-language-processing*[tiab] OR NLP[tiab]) OR ("Social Determinants of Health"[Majr] OR economic*[tiab] OR education*[tiab] OR employ*[tiab] OR income*[tiab] OR job[tiab] OR jobs[tiab] OR neighborhood*[tiab] OR poverty[tiab] OR resource*[tiab] OR SDOH[tiab] OR socio-economic*[tiab] OR SES[tiab] OR structural-determinant*[tiab] OR support*[tiab] OR work*[tiab]))

## 4. Conclusion

The ten candidate best papers of 2024 underscore a pivotal inflection point in HIE/HIS – a shift in focus from the mere capability to connect toward the tangible consequences of connection. This body of work reveals that technical progress alone is insufficient to realize the full promise of interoperability. Instead, success hinges on addressing enduring challenges in data quality, fragmentation, human-centered design, governance, and health equity. Important to the future of the international landscape, but outside of the 2024 paper review, is the March 2025 initiative by the European Commission to establish the European Health Data Space (EHDS) Regulation to foster interoperability through a harmonized legal and technical framework [ 22 ]. Pushing this framework forward, the European Commission further considers how generative AI can contribute to healthcare interoperability, especially since the European Union includes 24 languages and a large amount of unstructured content (June 2025) [ 22 ].

**Table:** 

Embase
(‘medical information system’/exp/mj OR ‘health information exchange*’:ab,ti OR ‘medical information exchange*’:ab,ti OR ‘clinical-information-system*or clinical-pharmacy-information-system*’:ab,ti OR ‘health information network*’:ab,ti OR ‘health information system*’:ab,ti OR ‘is h med’:ab,ti OR ‘medical information service*’:ab,ti) AND [2024-2024]/py AND (‘article’/it OR ‘article in press’/it OR ‘review’/it) AND ((‘artificial intelligence’/exp/mj OR ‘computer security’/exp/mj OR ‘medical informatics’/exp/mj OR ‘natural language processing’/exp/mj OR AI:ab,ti OR artificial-intelligence:ab,ti OR clinical-informatics:ab,ti OR computational-intelligence:ab,ti OR computer-reasoning:ab,ti OR computer-security:ab,ti OR computer-vision-system*:ab,ti OR cyber-security:ab,ti OR cybersecurity:ab,ti OR health-informatics:ab,ti OR Health-Information-Technolog*:ab,ti OR HIT:ab,ti OR machine-intelligence:ab,ti OR machine-learning:ab,ti OR medical-computer-science*:ab,ti OR medical-informatics:ab,ti OR medical-information-science*:ab,ti OR natural-language-processing*:ab,ti OR NLP:ab,ti) OR (‘social determinants of health’/exp/mj OR economic*:ab,ti OR education*:ab,ti OR employ*:ab,ti OR income*:ab,ti OR job:ab,ti OR jobs:ab,ti OR neighborhood*:ab,ti OR poverty:ab,ti OR resource*:ab,ti OR SDOH:ab,ti OR socio-economic*:ab,ti OR SES:ab,ti OR structural-determinant*:ab,ti OR support*:ab,ti OR work*:ab,ti))

Despite meaningful advances in connectivity infrastructure both domestically and internationally, the persistent tension between promise and practice is evident. Interoperability remains hindered by structural fragmentation, poor usability, and regulatory ambiguity. Data completeness, while technically achievable, is still undermined by inconsistent governance, inadequate workflows, and misaligned incentives [ 12 13 14 15 19 ]. Human factors continue to impact system use, with usability and documentation burdens detracting from clinical engagement and efficiency [ 10 12 17 18 20 ]. Governance frameworks, although maturing, must evolve beyond voluntary participation and fragmented coordination to include coherent, enforceable policies that promote integration across domains [ 14 15 19 ]. Importantly, the dimension of health equity, long under-explored in HIS, emerges as a critical consideration. These studies suggest that disparities in access and data representation risk being perpetuated, or even exacerbated, if equity is not made a design and implementation imperative [ 12 16 , 18 20 ].

Taken together, the findings from 2024 emphasize that technical interoperability is a necessary, but not sufficient, condition for impactful HIE/HIS. Advancing toward the next phase of system evolution will require a systemic rethinking of workflows, stakeholder alignment, regulatory harmonization, and equity-driven design. At this critical juncture, the opportunity is not just to connect systems—but to connect meaningfully, with measurable, equitable outcomes for patients, providers, and populations alike. Looking forward, our analysis suggests three critical priorities for the field. First, governance frameworks must evolve from voluntary participation models to more structured approaches that balance innovation with accountability. Second, interoperability efforts must extend beyond technical standards to address the organizational and cultural barriers that perpetuate data silos, particularly in behavioral health and public health domains. Third, implementation strategies must explicitly incorporate equity considerations, ensuring that advances in HIE/HIS benefit all populations rather than widening the digital divide. Lastly, the advances of AI – generative or otherwise – must be considered as powerful enablers to propel HIE/HIS utilization and use to levels beyond exchange, but rather toward discovery.

**Figure 1.**
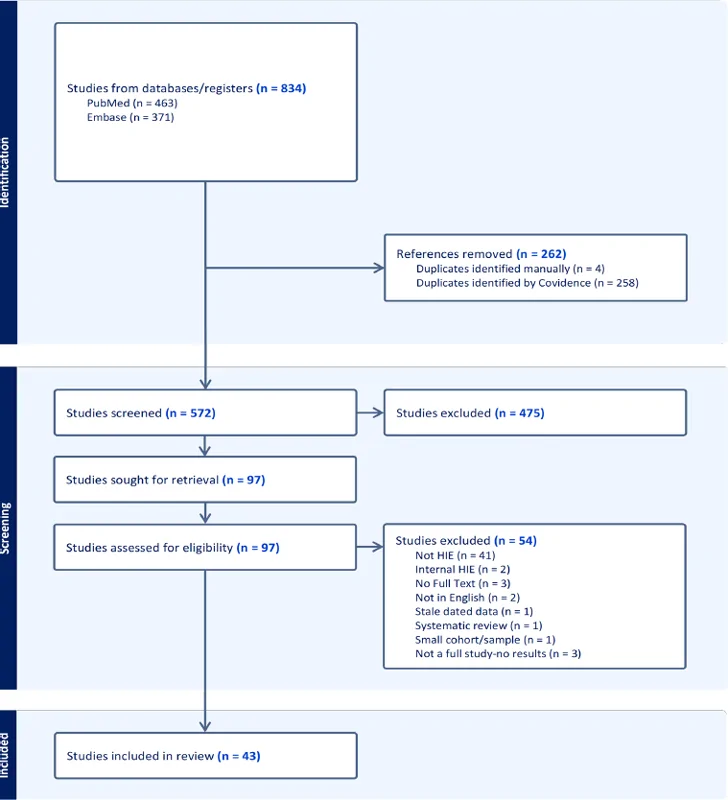
PRISMA diagram of literature search (Generated from Covidence.org )

## References

[1] Bloomrosen M, Berner ES. Findings from the 2023 Yearbook Section on Health Information Exchange. Yearb Med Inform, 2023. 32:195-200.https://doi.org/10.1055/s-0043-1768755 10.1055/s-0043-1768755 PMC10751133 38147861

[2] World Health Organization, Strategy for optimizing national routine health information systems: strengthening routine health information systems to deliver primary health care and universal health coverage, World Health Organization, 2024. URL:https://books.google.com/books/about/Strategy_for_Optimizing_National_Routine.html?id=F5EOEQAAQBAJ&printsec=frontcover&source=kp_read_button&hl=en&newbks=1&newbks_redir=1

[3] Bernstam EV, Warner JL, Krauss JC, Ambinde E, Rubinstein WS, Komatsoulis G, *et al.* Quantitating and assessing interoperability between electronic health records. J Am Med Inform Assoc, 2022. 29:753-760. https://doi.org/10.1093/jamia/ocab289 10.1093/jamia/ocab289 PMC9006690 35015861

[4] Brailer DJ. Interoperability: The Key To The Future Health Care System. Health Affairs, 2005.https://doi.org/10.1377/hlthaff.W5.19 10.1377/hlthaff.w5.19 15659454

[5] Middleton B. The Value of Healthcare Information Exchange and Interoperability, in: Healthcare Information Management Systems Society (HIMSS), Orlando, FL, 2004. URL:https://digital.ahrq.gov/sites/default/files/docs/library/CCHL_BPG.pdf

[6] Kaelber DC, Bates DW. Health information exchange and patient safety. J Biomed Inform, 2007. 40:S40-45.https://doi.org/10.1016/j.jbi.2007.08.011 10.1016/j.jbi.2007.08.011 17950041

[7] Feldman SS, Schooley BL, Bhavsar GP. Health information exchange implementation: lessons learned and critical success factors from a case study. JMIR Med Inform, 2014. 2:e19.https://doi.org/10.2196/medinform.3455 10.2196/medinform.3455 PMC4288070 25599991

[8] Enos G. Coordination through HIE allows MH agency to maintain client contact. Mental Health Weekly, 2023. 33:1-7.https://doi.org/10.1002/mhw.33757

[9] Organisation for Economic Cooperation and Development, Health at a Glance 2023: OECD Indicators. URL:https://www.oecd.org/content/dam/oecd/en/publications/reports/2023/11/health-at-a-glance-2023_e04f8239/7a7afb35-en.pdf

[10] Vest JR, Kampman H, Ben-Assuli O. Advancing Health Information Exchange: AI/ML, Social Needs, and Cybersecurity Trends. Yearb Med Inform, 2025 (to update) 10.1055/s-0046-1816622 PMC13730668 42976803

[11] Holmgren AJ, Adler-Milstein J, Apathy NC. Electronic Health Record Documentation Burden Crowds Out Health Information Exchange Use By Primary Care Physicians: Article examines electrnoic health record documentation burden. Health Aff, 2024. 43:1538-1545.https://doi.org/10.1377/hlthaff.2024.00398 10.1377/hlthaff.2024.00398 39496090

[12] DeLone WH, McLean ER. Information systems success: the quest for the dependent variable. Inf Syst Res, 1992. 3:60-95.https://doi.org/10.1287/isre.3.1.60

[13] Bhatt PR, Bhandari R, Adhikari S, Gahatraj NR. Health professionals' experience on district health information system (DHIS2) and its utilization at local levels in Gandaki province, Nepal: a qualitative study. PLOS Glob Public Health, 2024. 4:e0002890.https://doi.org/10.1371/journal.pgph.0002890 10.1371/journal.pgph.0002890 PMC10971587 38536828

[14] Coelho R, Rocha R, Hone T. Improvements in data completeness in health information systems reveal racial inequalities: longitudinal national data from hospital admissions in Brazil 2010-2022. Int J Equity Health, 2024. 23:143.https://doi.org/10.1186/s12939-024-02214-3 10.1186/s12939-024-02214-3 PMC11256545 39026324

[15] Dixon BE, Apathy NC. Interoperability in the Wild: Comparison of Real-World Electronic C-CDA Documents from Two Sources, in: MEDINFO 2023—The Future Is Accessible, IOS Press, 2024, pp. 43-47.https://doi.org/10.3233/SHTI230924 10.3233/SHTI230924 38269762

[16] Ha T, Kang S, Yeo NY, Kim TH, Kim WJ, Yi BK, *et al.* Status of MyHealthWay and Suggestions for Widespread Implementation, Emphasizing the Utilization and Practical Use of Personal Medical Data. Healthc Inform Res, 2024. 30:103-112. https://doi.org/10.4258/hir.2024.30.2.103 10.4258/hir.2024.30.2.103 PMC11098772 38755101

[17] Matthews EB. The Impact of Delivery Reform on Health Information Exchange with Behavioral Health Providers: Results from a National Representative Survey of Ambulatory Physicians. Adm Policy Ment Health, 2024. 51:818-825.https://doi.org/10.1007/s10488-024-01367-1 10.1007/s10488-024-01367-1 38512556

[18] Snyder ME, Nguyen KA, Patel H, Sanchez SL, Traylor M, Robinson MJ, *et al.* Clinicians' use of Health Information Exchange technologies for medication reconciliation in the US Department of Veterans Affairs: a qualitative analysis. BMC Health Serv Res, 2024. 24:1194. https://doi.org/0.1186/s12913-024-11690-w 10.1186/s12913-024-11690-w PMC11460093 39375765

[19] Suzumoto J, Mori Y, Kuroda T. Health Care Worker Usage of Large-Scale Health Information Exchanges in Japan: User-Level Audit Log Analysis Study. JMIR Med Inform, 2024. 12:e56263.https://doi.org/10.2196/56263 10.2196/56263 PMC11481819 39382566

[20] Everson J, Chang W, Patel V, Adler-Milstein J. The state of health information organizations and plans to participate in the federal exchange framework. Health Aff Sch, 2024. 2:qxae098.https://doi.org/10.1093/haschl/qxae098 10.1093/haschl/qxae098 PMC11346357 39188926

[21] Sasie SD, Van Zuylen P, Ayano G, Aragaw FM, Spigt M. Information sharing across institutions: Practices and barriers during public health emergencies in Ethiopia. Int J Med Inform, 2024. 186:105439.https://doi.org/10.1016/j.ijmedinf.2024.105439 10.1016/j.ijmedinf.2024.105439 38564958

[22] European Commission, European Health Data Space Regulation (EHDS), What is the EHDS Regulation about. URLhttps://health.ec.europa.eu/ehealth-digital-health-and-care/european-health-data-space-regulation-ehds_en

